# Elevated serum leptin levels are associated with lower renal function among middle-aged and elderly adults in Taiwan, a community-based, cross-sectional study

**DOI:** 10.3389/fendo.2022.1047731

**Published:** 2022-12-23

**Authors:** Yu-Lin Shih, Chin-Chuan Shih, Sun-Yi-Fan Chen, Jau-Yuan Chen

**Affiliations:** ^1^ Department of Family Medicine, Chang-Gung Memorial Hospital, Taoyuan, Taiwan; ^2^ General Administrative Department, United Safety Medical Group, New Taipei, Taiwan; ^3^ College of Medicine, Chang Gung University, Taoyuan, Taiwan

**Keywords:** leptin, renal function, chronic kidney disease, adipocyte, middle-aged and elderly, obesity

## Abstract

**Background:**

Plasma leptin is considered a risk factor for obesity and cardio-metabolic disease, but the link between serum leptin and renal function is still under evaluation. In our study, we focused on the relationship between serum leptin and renal function, and we investigated the relationship in more detail.

**Methods:**

The 396 middle-aged and elderly Taiwanese adults recruited for our health survey were the subject of our research. All participants agreed to participate and signed a consent form before they joined and completed our study. We divided the participants into three groups according to eGFR tertiles and analyzed the parameters between each group. Then, we used Pearson’s correlation test to investigate the relationship between eGFR levels and cardio-metabolic risk factors with adjustment for age. The scatter plot indicates the trend between serum leptin levels and eGFR levels. Participants were reclassified into three subgroups according to their leptin levels and the bar chart reveals the prevalence of chronic kidney disease (CKD) in each group. Finally, we used multivariate linear regression to evaluate the relationship between serum leptin and eGFR levels with adjustment for age, sex, smoking status, drinking status, body mass index (BMI), uric acid levels, hypertension (HTN), diabetes mellitus (DM), and dyslipidemia.

**Results:**

In our study, we analyzed the data from 396 eligible participants. A total of 41.4% of the participants were male, and the average age of all participants was 64.81 years ( ± 8.78). The participants in the high eGFR group were more likely to have lower serum leptin levels. Furthermore, eGFR values were negatively correlated with serum leptin levels even after adjustment for age. The prevalence of CKD in the high serum leptin group was higher than that in the low serum leptin group. Serum leptin levels showed significant negative correlations with eGFR levels (β=-0.14, p<0.01) in the multivariate linear regression after adjusting for age, sex, smoking status, drinking status, BMI, uric acid levels, HTN, DM, and dyslipidemia.

**Conclusion:**

According to our study, serum leptin levels show a negative relationship with eGFR levels in middle-aged and elderly people in Taiwan. In addition, high serum leptin levels could be an novel marker to survey kidney failure in clinical practices.

## 1 Introduction

Obesity has a high prevalence in modern society (1). Many chronic diseases, such as cardiovascular diseases or metabolic syndrome, are linked to obesity, and the mechanisms between these chronic diseases and obesity have been described in previous studies (2, 3). On the other hand, CKD is another heavy burden on the health system, especially when patients enter end-stage renal disease. CKD has been considered a risk factor for other chronic diseases, such as coronary artery disease (4). Obesity also has a negative effect on renal function. Studies have revealed that obesity can compromise renal function (5).

Leptin is a small peptide hormone that is mainly released by adipocyte tissue and encoded by the obese (ob) gene (6). The discovery of leptin increases the importance of adipocytes, which were only previously considered as energy storing cells. With leptin, adipocytes play a crucial role in endocrinology (7). Leptin mainly acts as negative feedback to suppress appetite and reduce fat storage (8). The basic physiological function of leptin is controlling energy balance in the body, and the leptin level is positively related to the amount of body adipocyte tissue (9). Moreover, the pathophysiological role of leptin was also revealed by recent studies. For example, leptin resistance can be found in many overweight patients and exacerbates their obesity, which leads to metabolic disorders (10). Moreover, many studies indicate the relationship between obesity and chronic kidney disease, but the pathophysiology between obesity and renal function impairment is underdeveloped (11). In addition, leptin is a small peptide that is cleared by the kidney, so renal function also has an impact on the homeostasis of plasma leptin (12). On the other hands, some studies indicated that leptin can elevate blood pressure (13), trigger inflammation (14), and increase glomeruloslcerosis (15). Those mechanism may explained the relationship between leptin levels and renal function. However, aging is an important risk factor for many chronic disease, including kidney failure. There is no research focus on age population, which is vulnerable to chronic kidney disease. In our community-based study, we investigated leptin, which is considered a biomarker of obesity, in association with CKD in middle-aged and elderly people in northern Taiwan. Our results can provide a potential direction for future research and a possible reference for primary care.

## 2 Materials and methods

### 2.1 Study design and participants

**Figure 1:**
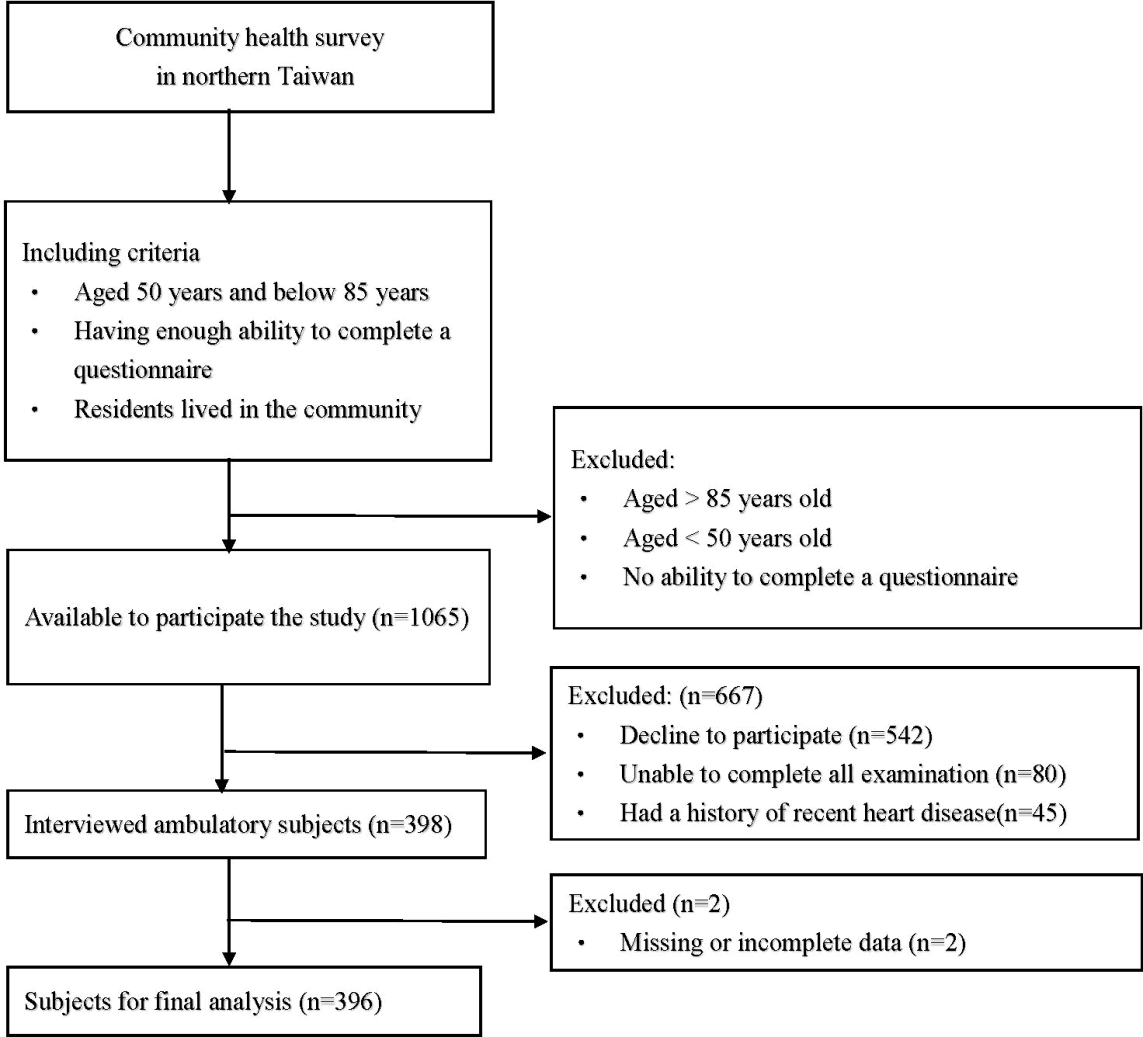
Flow chart of study subjects.

This was a cross-sectional and community-based health survey project conducted in 2019 in northern Taiwan, and the participants were all from this project. The health survey project was held in several clinics in different communities, and we chose the candidates in a consecutive manner. Then, the participants were selected from those candidates under including and excluding criteria. The inclusion criteria were as follows: (1) individuals who agreed to participate in the study (2) individuals who lived in the community; (3) individuals between 50 and 85 years of age; (4) individuals who were able to complete a questionnaire; and (5) individuals who can complete examinations. The exclusion criteria were (1) a history of recent heart disease; (2) missing or incomplete data; (3) failing to finish the examination or questionnaire, or (4) withdrawal during the study. The flowchart which we used to select the participants is presented in **Figure 1**. Every participant was informed and signed an informed consent form before they entered the study. Ultimately, a total of 396 subjects were recruited for our study. All of the participants completed the questionnaire during a face-to-face interview and provided blood and urine samples. This study was approved by the Chang Gung Medical Foundation Institutional Review Board (IRB) (IRB No.: 201801803B0).

### 2.2 Data collection and laboratory measurements

The content of the questionnaire included sex, age, drinking status, smoking status, body weight, and body height. The health survey collected hypertension (HTN), diabetes mellitus (DM), and dyslipidemia data. Resting systolic blood pressure (SBP, mmHg) and diastolic blood pressure (DBP, mmHg) were measured at rest at least two times. The following biochemical laboratory parameters were analyzed at the Roche^®^ model lab at Taiwan E&Q Clinical Laboratory: leptin (ng/mL), estimated glomerular filtration rate (eGFR, ml/min/1.73 m2), creatinine (mg/dl), uric acid (mg/dl), fasting plasma glucose (FPG, mg/dl), and triglycerides (mg/dl). The leptin level (ng/mL) was analyzed by Enzyme-linked Immunosorbent Assay using Invitrogen™ Human Leptin. Body mass index (BMI) was calculated as the person’s weight in kilograms divided by the square of their height in meters.

### 2.3 Definitions of CKD, DM, HTN and dyslipidemia

CKD was defined as the presence of kidney damage (urine ACR ≥30 mg/g) or decreased renal function with an eGFR <60 mL/min/1.73 m2 (31) (16). DM was defined as a fasting plasma glucose level ≥126 mg/dL or the use of oral hypoglycemic agents or insulin therapy (17). HTN was defined as an SBP ≥140 mmHg, a DBP ≥90 mmHg, or the use of treatment for HTN (18). Dyslipidemia was defined as an LDL-C level ≥130 mg/dL, an HDL-C level <40 mg/dL in men or <50 mg/dL in women, a TG level ≥150 mg/dL, a TC level ≥200 mg/dL, or the use of lipid-lowering medication (19).

### 2.4 Statistical analysis

Participants were divided into three groups according to eGFR level: a low eGFR level group (eGFR < 78.2 ml/min/1.73 m2), middle low eGFR level group (78.2 ml/min/1.73 m2 ≤ eGFR ≤ 94.3 ml/min/1.73 m2), and high eGFR level (94.3 ml/min/1.73 m2 < eGFR) group. In **Table 1**, we used Shapiro-Wilk normality test to check the normality of continuous data. The continuous variables that were consistent with normal distribution were presented as mean ± [SD], and the p-values were calculated by one-way ANOVA; the continuous variables that did not conform with normal distribution were presented as median (Q1, Q3), and the p-values were derived from Kruskal-Wallis ANOVA; categorical variables are expressed as n (%) and were analyzed by the chi-square test. Pearson’s correlation coefficient was used to analyze correlations between eGFR levels and leptin levels, age, FPG levels, triglyceride levels, SBP, BMI, and uric acid levels; Pearson’s correlation adjusted by age was also performed. The scatter plot illustrates the trend between eGFR and serum leptin levels. In addition, we further calculated the prevalence of CKD according to the three levels of leptin. Finally, a multivariate linear regression was performed to evaluate the association between leptin and eGFR levels after adjusting for age, sex, smoking status, drinking status, BMI, uric acid levels, HTN, DM, and dyslipidemia. In our study, a p value of < 0.05 was considered statistically significant. All statistical analyses were performed using SPSS for Windows (IBM Corp. Released 2011. IBM SPSS Statistics, version 25.0. Armonk, NY: IBM Corp.)

**Table 1:** General characteristics of the study population according to tertiles of eGFR.

	eGFR				
variable	total	Low	middle	high	
		(<78.2)	(78.2~94.3)	(>94.3)	
	(n=396)	(n=132)	(n=133)	(n=131)	P value
Leptin (ng/mL)	14.89 (8.49, 28.36)	16.72 (9.45, 39.65)	15.01 (8.67, 26.60)	13.31 (7.74, 21.67)	0.021
eGFR (ml/min/1.73m^2^)	87.13 ± 22.40	64.14 ± 12.26	85.93 ± 4.69	111.51 ± 14.61	<0.001
Creatinine (mg/dl)	0.80 (0.68, 0.97)	1.06 (0.87, 1.20)	0.77 (0.72, 0.94)	0.64 (0.58, 0.78)	<0.001
Age (year)	64.81 ± 8.78	68.90 ± 8.93	64.72 ± 8.26	60.77 ± 7.16	<0.001
Uric Acid (mg/dl)	5.63 ± 1.52	6.23 ± 1.71	5.51 ± 1.31	5.16 ± 1.30	<0.001
BMI (kg/m^2^)	25.59 ± 3.84	26.12 ± 3.92	25.62 ± 4.02	25.04 ± 3.51	0.023
FPG (mg/dl)	99.00 (89.00, 118.75)	100.00 (90.00, 122.75)	100.00 (90.00, 116.00)	97.00 (89.00, 119.00)	0.526
Triglyceride (mg/dl)	118.00 (86.00, 165.00)	130.00 (88.25, 171.75)	111.00 (82.50, 156.50)	112.00 (86.00, 167.00)	0.146
SBP(mmHg)	137.30 ± 17.49	140.11 ± 19.39	137.79 ± 17.41	133.98 ± 14.94	0.004
DBP(mmHg)	85.19 ± 10.98	85.59 ± 11.88	85.11 ± 10.15	84.86 ± 10.92	0.592
Gender, male (%)	164 (41.4%)	59 (44.7)	53 (39.8)	52 (39.7)	0.410
Smoking (%)	50 (11.7%)	13 (9.8%)	10 (7.5%)	27 (20.6%)	0.090
Drinking (%)	28 (6.6%)	5 (3.8%)	6 (4.5%)	17 (13.0%)	0.004
HTN, n (%)	201 (50.8%)	88 (66.7)	62 (46.6)	51 (38.9)	<0.001
DM, n (%)	133 (33.6%)	55 (41.7)	39 (29.3)	39 (29.8)	0.041
dyslipidemia, n (%)	153 (38.6%)	57 (43.2)	45 (33.8)	51 (38.9)	0.478

continuous variables that were consistent with normal distribution were presented as mean ± [SD], and the p-values were calculated by one-way ANOVA; the continuous variables that did not conform with normal distribution were presented as median (Q1, Q3), and the p-values were derived from Kruskal-Wallis ANOVA. The categorical variables were presented as n(%), and chi-square test were performed for the p-values.

eGFR, estimated glomerular filtration rate; BMI, body mass index; FPG, fasting plasma sugar; SBP, systolic blood pressure; DBP, diastolic blood pressure; HTN, hypertension; DM, diabetes mellitus.

## 3 Results

The data were collected from middle-aged and elderly people from communities in northern Taiwan. A total of 396 individuals, including 164 (41.4%) men and 232 (58.6%) women with a mean age of 64.81 ± 8.78 years, were enrolled for analysis. **Table 1** demonstrates the demographic and clinical characteristics of our study group. The participants were categorized into one of three subgroups according to their eGFR: a low eGFR group (<78.2 ml/min/1.73 m^2)^, middle eGFR group (78.2~94.3 ml/min/1.73 m^2)^, and high eGFR group (>94.3 ml/min/1.73 m^2)^. The average eGFR and leptin levels of our study group were 87.13 ± 22.40 ml/min/1.73 m^2^ and 14.89 (8.49, 28.36) ng/ml, respectively. There was no statistically significant difference in FPG levels, triglyceride levels, DBP, sex, or dyslipidemia among the low, middle, and high eGFR subgroups. The participants in the low eGFR group were more likely to have a higher leptin concentration, creatinine concentration, age, uric acid concentration, BMI, SBP, possibility of HTN, and possibility of DM.

**Figure 2:**
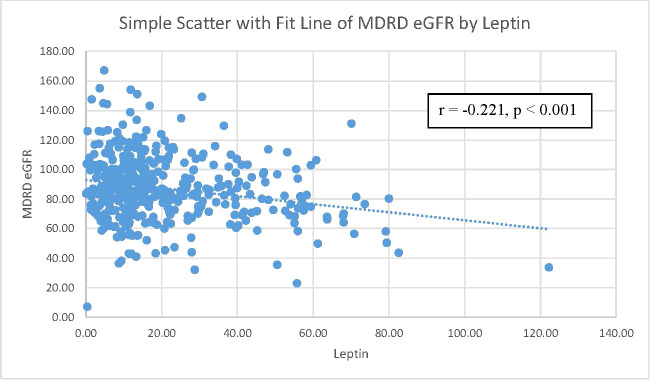
The correlation between MDRD eGFR and leptin level. Notes: a scatterplot of MDRD eGFR by leptin level. The Pearson’s correlation coefficient was 0.221 with a p-value=0.001. MDRD eGFR, Modification of Diet in Renal Disease Study equation estimated glomerular filtration rate.

**Table 2** further shows the correlations between eGFR levels and various cardio-reno-metabolic risk factors with Pearson’s correlation. eGFR levels were negatively correlated with leptin levels, age, SBP, BMI, and uric acid levels. eGFR levels did not have a significant relationship with FPG or triglyceride levels in our study. However, only leptin levels, BMI, and uric acid levels remained statistically significant after adjusting for age. **Figure 2** shows the relationship between leptin levels and eGFR levels. Pearson’s correlation coefficient was -0.221 with a p value<0.001.

**Table 2:** The Pearson’s Correlation between eGFR and serum leptin level.

variable	eGFR			
	Unadjusted		Adjusted for age	
	Correlationcoefficient	P vale	Correlationcoefficient	P vale
Leptin (ng/mL)	-0.221	<0.001	-0.192	<0.001
Age (year)	-0.387	<0.001	NA	NA
FPG (mg/dl)	-0.064	0.201	-0.078	0.120
Triglyceride (mg/dl)	0.066	0.190	0.033	0.507
SBP(mmHg)	-0.165	<0.001	-0.091	0.072
BMI (kg/m^2^)	-0.115	0.023	-0.150	0.003
Uric Acid (mg/dl)	-0.346	<0.001	-0.366	<0.001

eGFR, estimated glomerular filtration rate; FBS, fasting blood sugar; SBP, systolic blood pressure; BMI, body mass index; NA, not available.

**Figure 3:**
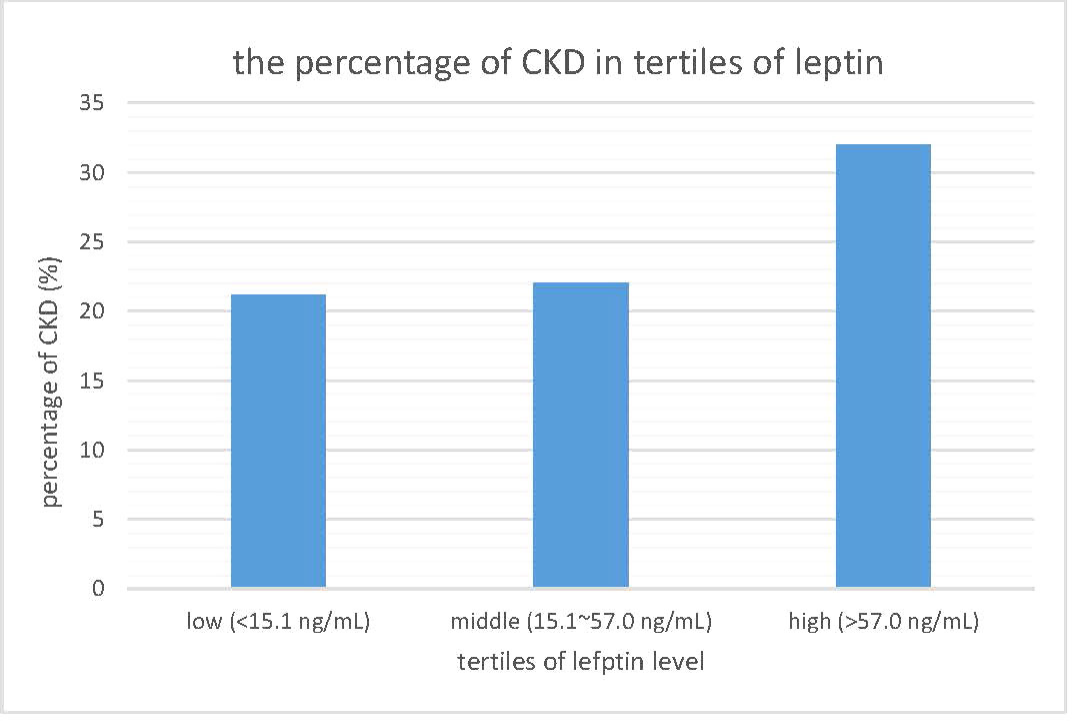
The prevalence of chronic kidney disease in tertiles of leptin level. CKD, chronic kidney disease.

To investigate the relationship between leptin levels and CKD, we reclassified the participants into three subgroups according to their leptin levels: a low leptin level group (<15.1 ng/mL), middle leptin level group (15.1~57.0 ng/mL), and high leptin level group (>57.0 ng/mL). Then, we calculated the percentage of CKD in each subgroup, and the results are shown in **Figure 3**. There was a linear increasing trend across leptin tertiles, with a p value for trend of 0.034. The prevalence of CKD risk was significantly greater in the groups with higher leptin levels. The negative relationship between renal function and leptin levels led us to speculate that there is an association between eGFR and leptin levels.

**Table 3** presents the association of eGFR and leptin levels, showing that leptin levels were significantly negatively correlated with eGFR levels in both simple and multiple linear regression models that were adjusted. The association persisted even after adjustment for age, sex, smoking status, drinking status, BMI, uric acid levels, HTN, DM, and dyslipidemia.

**Table 3:** Association between leptin and eGFR in multivariate linear regression.

	Model 1			Model 2			Model 3		
	β	S.E.	P value	β	S.E.	P value	β	S.E.	P value
leptin	-0.18	0.04	<0.001	-0.13	0.034	<0.001	-0.14	0.034	<0.001

model 1: unadjusted.

model 2: adjusting for model 1 plus age, gender, smoking, drinking, BMI, and uric acid.

model 3: adjusting for model 2 plus HTN, DM, and dyslipidemia.

BMI, body mass index; HTN, hypertension; DM, diabetes mellitus.

## 4 Discussion

The eGFR indicates the ability of the kidneys to remove toxins and metabolites, including uric acid and creatinine, which also act as parameters in calculating eGFR (20). A low eGFR will elevate serum creatinine and uric acid. In **Table 1**, we also found elevated plasma creatinine and uric acid levels in the high plasma leptin group. From a previous medical study, renal function declined steadily with age (21), and our study showed a similar result: the low eGFR group tended to be older. Obesity is another risk factor for renal function impairment (22). There is much research describing how obesity compromises renal function, including lipid toxicity (23), inflammatory stress (24), adipokines (25), and oxidative stress (26). Our research also showed a higher BMI in the low eGFR group. High blood pressure elevates intercapillary flows and causes progressive glomerular damage, followed by *in situ* inflammation or even microthrombosis. Eventually, elevated blood pressure decreases eGFR levels (27). As shown in **Table 1**, the lower eGFR group tended to have a higher prevalence of HTN and higher SBP. Our result corresponds with previous studies. DM is another risk factor for renal function impairment (28). As shown in **Table 1**, the low eGFR group tends to have higher prevalence of DM and this result was mentioned in previous studies, which demonstrates the loss of podocytes, basement membrane disruption and glomerular sclerosis in DM patients (29). Importantly, we noted a statistically significant increase in serum leptin levels in the low eGFR group. This result evoked our curiosity about the relationship between serum leptin levels and renal function.

**Table 2** focuses on the relationship between eGFR and other parameters. Pearson’s correlation coefficients are shown in **Table 2**. eGFR levels were negatively correlated with serum leptin levels, age, SBP, BMI, and uric acid levels. Among these parameters, only serum leptin levels, BMI, and uric acid levels still remained significantly negatively related to eGFR levels. The scatter plot in **Figure 2** demonstrates the Pearson’s correlation between serum leptin levels and eGFR levels with a Pearson’s correlation coefficient of -0.221 and a p value <0.001. As shown in **Figure 3**, we divided participants into three subgroups according to their leptin level: the low leptin level group (<15.1 ng/mL), middle leptin level group (15.1~57.0 ng/mL), and high leptin level group (>57.0 ng/mL). We found that the high serum leptin group tended to have a higher prevalence of CKD. Based on our previous findings, we explored the relationship between eGFR and serum leptin levels while considering other parameters.

As we mentioned in **Table 1**, there are many parameters that can affect eGFR levels, including creatinine levels, age, uric acid levels, BMI, SBP, HTN, and DM. Obesity has a strong relationship with DM (30), and excessive fat tissue will lead to the secretion of more leptin (31). Taking these relationships into consideration, multivariate linear regression was used (see **Table 3**) to weight the relationship between eGFR and serum leptin levels with the adjustment of other parameters. After adjusting for age, sex, smoking status, drinking status, BMI, uric acid levels, HTN, DM, and dyslipidemia, serum leptin levels and eGFR levels were still negatively correlated, with a regression coefficient of -0.14 and p value <0.001.

Leptin is a small peptide hormone that is mainly produced by adipocytes and regulates energy homeostasis (32). Moreover, leptin also regulates other physiological functions in peripheral tissue, such as the release of other neurotransmitters (33), the modulation of insulin function (34) and angiogenesis (35), and the modification of the immune system (36). Recent studies also revealed that leptin can be produced by other tissues, including the stomach, skeletal muscles, pituitary gland, and mammary gland (37). The kidney is responsible for leptin clearance (12), and leptin contributes to the pathophysiology of the kidney. Accumulating data indicate that leptin has direct and indirect effects on the kidney, which may deteriorate renal function.

Leptin can elevate sympathetic nervous activity. In an animal model, mice that overexpressed leptin had high blood pressure and increased catecholamine excretion in their urine. Previous research found that the neuron that secretes proopiomelanocortin is also a target of leptin (37). α-melanocyte-stimulating hormone and β-endorphin, which may activate sympathetic activity and mean arterial pressure, are both derived from proopiomelanocortin (38). Hypertension is a risk factor for CKD. Prolonged high blood pressure damages the endothelium and eventually causes glomerulosclerosis, which leads to compromised renal function (39). Indeed, we observed high systolic blood pressure and elevated leptin levels in the low eGFR group.

The kidney is also affected by leptin directly. Further investigation revealed that leptin can induce the messenger ribonucleic acid (mRNA) of transforming-factor-β1 (TGF-β1) and increase the secretion of TGF-β1 in glomerular endothelial cells. Leptin also amplifies the expression of TGF-β1 type II receptors and sensitizes mesangial cells to TGF-β1 (40). Then, TGF-β1 from the glomerular endothelium acts in a paracrine manner to increase the production of collagen from sensitized mesangial cells. Excessive collagen deposition in the extracellular matrix may disrupt the function of the glomerulus and eventually contribute to proteinuria and glomerulosclerosis (41).

Leptin also triggers the inflammatory process, and recent studies have also indicated the regulatory function of leptin on the immune response. Leptin can increase proinflammatory cytokines, including interleukin-1 (IL-1), tumor necrosis factor-α (TNF-α), and interleukin-6 (IL-6) (42, 43). These proinflammatory cytokines in turn trigger adipocytes to secrete more leptin and proinflammatory cytokines, such as TNF-α (44). On the other hand, leptin also regulates T-cell immunity by increasing Th1 cytokine production and suppressing Th2 cytokine production (45). These immunological stimuli cause chronic inflammation in patients with hyperleptinemia. Eventually, the proinflammatory status compromises renal function.

In recent studies, leptin demonstrated the ability to induce the intracellular accumulation of reactive oxygen species (ROS). Leptin causes intracellular accumulation of reactive oxygen species (ROS) (46), which are associated with activation of the JNK/SAPK-dependent pathway and the redox-sensitive transcription factor NF-κB (47). This consequence indicates that leptin plays a major role in the accumulation of ROS in endothelial cells. It is possible that the hyperleptinemia may also trigger renal oxidative stress and leads to kidney damage.

There are several novelties in our research. First, aging is the main risk factor for many chronic diseases, including kidney failure, and the aged populations are vulnerable to kidney failure (48). Previous studies found the relationship between leptin levels and chronic kidney diseases, but we focused on the vulnerable populations, the middle-aged and elderly adults. Plus, we recruited the participants from the community, so our result can truly represent the situation of the middle-aged and elderly population in the community. Our result provide useful information for disease prevention and screening in clinical practices. Second, we designed our research with clear outlet, sufficient sample size, enough and important confounders, and meticulous data analysis. However, our study still had several limitations. Regarding the medication, although we recorded the medication of the participants, many participants can not totally recall their medication, including analgesics. This shortcoming compromised our medication data, so we excluded the data on medication to preserve the integrity of the data during the analysis. Another common limitation is that our participants were all selected from northern Taiwan, so the selection bias should be aware. Future research which uses larger population and includes the medication would make the result more robust.

## 5 Conclusion

According to our study, serum leptin levels show a negative relationship with eGFR levels in middle-aged and elderly people in Taiwan. In addition, high serum leptin levels could be an novel marker to survey kidney failure in clinical practices.

## Data availability statement

The raw data supporting the conclusions of this article will be made available by the authors, without undue reservation.

## Ethics statement

The studies involving human participants were reviewed and approved by Chang Gung Medical Foundation Institutional Review Board. The patients/participants provided their written informed consent to participate in this study.

## Author contributions

Y-LS and J-YC composed and conducted the study. C-CS helped with data collection. J-YC provided instruction and consultation. Project administration: J-YC and Y-LS. Y-LS processed and analyzed the data. Y-LS and S-Y-FC finished academic writing. Y-LS completed the publication process. All authors contributed to the article and approved the submitted version.
